# Evaluation of the efficacy and tolerability of levetiracetam as add-on therapy in intractable epilepsy of children

**DOI:** 10.22037/ijcn.v15i4.29028

**Published:** 2022-03-14

**Authors:** Razieh Razieh, Alireza SHAFIEI, Fatemah DEHGHANI FIROUZABADI, Ali FATHI

**Affiliations:** 1 Pediatric Neurologist, Department of Pediatrics, Children Growth Disorder Research Center, Shahid Sadoughi University of Medical Sciences, Yazd, Iran; 2 Allergist and Clinical Immunologist, Assistant professor, Division of Allergy and Clinical Immunology, Department of Pediatrics, Bahrami Hospital, Tehran University of Medical Sciences,Tehran, Iran.; 3 General Physician, Shahid Sadoughi University of Medical Sciences, Yazd, Iran; 4 General Physician, Tehran University of Medical Sciences, Tehran, Iran

**Keywords:** Levetiracetam, Refractory Epilepsy, Children, Side effects

## Abstract

**Objective:**

One-third of epilepsy of children is refractory, and this study was conducted to evaluate the efficacy and adverse events of levetiracetam as add-on therapy in the treatment of refractory epilepsy of children.

**Materials & Methods:**

In this quasi-experimental study, seizures frequency and side effects of 314 children aged 1-14 years with refractory epilepsy were referred to the Pediatric Neurology Clinic of Shahid Sadoughi Medical Sciences University, Yazd, Iran, and treated with levetiracetam for six months, were evaluated.

**Results:**

We evaluated 142 girls and 172 boys with a mean age of 6.78±2.12 years. At the end of six months of treatment with levetiracetam, 20% became seizure-free, 28% had more than 50% decrease in seizure frequency, 38% did not have a notable change in seizure frequency, and 14% experienced an increase in seizure frequency. Good response (stopping of all seizures or more than 50% reduction in seizure frequency) was seen in 51% of mixed types, 61% of myoclonic seizures, 64% of generalized tonic-clonic seizures, 69 % of partial seizures, 100 % of tonic seizures, and in 40 % of atonic seizures. Levetiracetam was significantly more effective in partial seizures, idiopathic epilepsies, and children with normal developmental status and normal brain MRI.

Twelve children discontinued the treatment due to severe drowsiness, restlessness, and exacerbation of seizures. Transient and mild side effects, including somnolence, anorexia, fatigue, headache, ataxia, and diplopia, were seen in 9% (N=28) of patients.

**Conclusion:**

Levetiracetam could be considered an efficient and safe adjunct therapy in treating refractory epilepsy in children.

## Introduction

Epilepsy as a brain disorder is defined based on " the International League Against Epilepsy (ILAE), by any of the following conditions: 

1. At least two unprovoked (or reflex) seizures occurring >24 h apart

2. One unprovoked (or reflex) seizure and a probability of further seizures similar to the general recurrence risk (at least 60%) after two unprovoked seizures, occurring over the next ten years

3. Diagnosis of an epilepsy syndrome" ^(^^1^^)^.

"The clinical diagnosis of epilepsy usually requires the occurrence of at least one unprovoked epileptic seizure with either a second such seizure or enough electroencephalogram (EEG) and clinical information to demonstrate an enduring predisposition to develop recurrences convincingly. For epidemiologic and, commonly, for clinical purposes, epilepsy is considered when two or more unprovoked seizures occur in a time frame of longer than 24 hr. The cumulative lifetime incidence of epilepsy is 3%, and the annual prevalence is 0.5–1.0%. " ^(^^2^^)^

Intractable or refractory epilepsy is defined by the ILAE as recurrence of at least one seizure in a week despite taking two or three appropriate antiepileptic drugs (AEDs) with sufficient dosage ^(^^3^^)^, which includes approximately one-third of newly treated patients with epilepsy.^ (^^4^^)^

Intractable seizures, on the one hand, may lead to more extensive brain insult and, on the other hand, result in frequent referring to neurologists, hospitalization, financial burden, and the negative impact on the quality of life of the affected child and his or her family. Complete elimination of seizures or effective control of seizures is necessary to reduce epilepsy-related morbidity and mortality, and new AEDs with greater efficacy and fewer side effects are fiercely needed. Levetiracetam (LEV) is one of the newest antiepileptic drugs that has entered the market since 2000 and has been approved by the Food and Drug Administration since 2006 and is currently available in pills of 250, 500, and 1000 mg and also syrup (100 mg/ ml) in our country. For metabolism, it does not enter the cytochrome P450 enzyme system and is excreted directly and unchanged from the kidney, and its dose adjustment in patients with kidney failure is inevitable. In children, well toleration of the drug, its linear pharmacokinetics and complete oral absorption, the low extent of metabolism, and no interaction with other AEDs, lead to low discontinuation rates. The most common reported side effects in children are behavioral symptoms (irritability, agitation, hyperactivity, and cognitive slowing), and CNS adverse events, such as somnolence, asthenia, and dizziness, are usually less than other AEDs. LEV side effects are reversible, mild, and transient. Its mechanism of action is not precisely understood and has multiple mechanisms, but possibly act through voltage-dependent sodium channels that lead to a suppressive effect on the presynaptic release of excitatory neurotransmitter (glutamate and aspartate) release, as well as an increase in the concentration of inhibiting neurotransmitter gamma-aminobutyric acid in the brain, reducing the flow with no apparent impact on the activity of NMDA glutamate receptors, and carbonic anhydrase of the erythrocyte. The effective dose in pediatric patients is 20-40 mg/kg/day, given in two or three divided doses; however, it may be given up to 60 mg/kg/day. There is no direct correlation between LEV blood levels and seizure control, the drug side effects, or administered dosage. Therefore, the measurement of blood levels of LEV does not seem to be helpful. ^(^^2^^, ^^5^^, ^^6^^)^

Its efficacy has substantially controlled generalized tonic-clonic, myoclonic, and partial seizures in children. ^(^^5^^-^^14^^) ^Given that children with refractory epilepsy represent a significant percentage of referral cases to pediatric neurology clinics and because LEV has lately been introduced in our country, this study was conducted with the primary objective of evaluation of the efficacy, safety, and tolerability of this medication as add-on therapy in the treatment of intractable pediatric epilepsy.

## Materials & methods

In this quasi-experimental study, all consecutive 1-14-year-old children diagnosed with intractable epilepsy based on history, physical examination, and clinical judgment of a pediatric neurologist who were referred to the Pediatric Neurology Clinic of Shahid Sadoughi Medical Sciences University, Yazd, Iran, from March 2014 to January 2019 treated with LEV for six months were enrolled.

Classification of seizure types, epileptic syndromes, and definition of drug-resistant epilepsy in this study was based on ILAE Commission on Therapeutic Strategies Consensus. ^(^^3^^, ^^15^^)^

Initially, the parents of the children who met the criteria to enter the study were interviewed by the researcher, and after receiving their consent of them, their children entered the study.

Care was taken to include epileptic children aged 1-14 years who had not responded to adequate dosage of at least two conventional antiepileptic drugs in mono or polytherapy, had at least one seizure in a week, had no allergy to LEV, had not used LEV formerly, and maintained on LEV for six months. 

In the baseline phase of the study, the average number of seizures per month in one month before registering into the study was recorded, and information on variables of the study, including age, sex, age of seizure onset, type and frequency of seizures (based on history), number of previous anticonvulsant drugs, type of epilepsy, etiologic classiﬁcation of epilepsy, and brain MRI and electroencephalographic results were reviewed. 

In the titration phase of the research, to minimize LEV clinical adverse effects, LEV was added to previous antiepileptic drugs in a four-week period as follows: 10, 20, 30, and 40 mg/kg/day in two divided doses and in the maintenance phase of the study, maximum dose or the dose, which controlled seizures maintained for six months. Clinical information about the type and frequency of seizures and clinical adverse effects of LEV were recorded by interviewing their parents. 

At the end of the period, the evaluation of LEV efficacy was made based on a comparison of the weekly frequency of major seizures of the patient in one month before and six months after LEV treatment.

Stopping of all seizures or reduction of more than 50% in the weekly frequency of seizure was considered as a good response to LEV, and also the following classiﬁcation was considered on this basis:

- Seizure-free: No more seizure attack was experienced.
- Improved: The patient's seizure frequency was reduced by more than 50%.
- Unchanged: No notable change was seen in seizure frequency.
- Worsened: seizure frequency increased more than 25%.

LEV serum levels were not checked in this research. Side effects of the medication were surveyed by a researcher via asking directly from the patient and, in case of a child having mental retardation, the questions were directed to the mothers. In case of non-attendance, phone follow-up was pursued with the availability of the patient's address and phone.

The data were analyzed using Statistical Package for the Social Sciences version 17 (SPSS, Chicago, IL, USA) statistical software. The Chi-square test was used to analyze qualitative variables, and mean values were compared using paired T-test. 

Informed consent was taken from children’s parents before the administration of the drug, and the study was approved by the Ethics Committee of Shahid Sadoughi University of Medical Sciences, Yazd, Iran, and the researchers received no support from the drug company. 

## Results

Three hundred thirty eligible children were enrolled in the study, but 16 patients were excluded from the study due to not returning or inadequate compliance and finally, 314 children, including 142 girls and 172 boys with a mean age of 6.78 ± 2.12 years, were evaluated and completed the entire course of the study. The age of onset of seizures was two months to 12.5 years, with a mean age of 2.01± 0.82 years. MRI was normal in 119 (38%), and 243 patients (77.3%) had developmental delay.

In this study, 106 children (33.7%) showed a mixed type of seizures (more than one type of seizure), and the major seizure type was myoclonic in 89 children (28.3%), generalized tonic-clonic in 59 (18.8%), partial in 29 (9.2%), tonic in 22 (7%) and atonic in nine (3%) children. Seizures were not controlled despite the usage of 5-14 antiepileptic drugs (mean ± SD: 7.4 ± 3.2). Based on the etiologic classiﬁcation of epilepsy, 43% (135) had symptomatic, 36% (114) cryptogenic, and 65 (21%) had idiopathic epilepsy. 

At the end of six months of LEV treatment, 20% (63 children) became seizure-free, 28% (88 children) had more than 50% reduction in seizures frequency, 38% (119 children) did not have a notable change in seizure frequency, and 14% (44 persons) experienced more than 25% increase in seizure frequency. 

 The mean dose of LEV for seizure control was 27.9 ± 5.4 mg/kg/day (range: 10-40). Good response (stopping of all seizures or more than 50% reduction in seizure frequency) was seen in 51% of mixed type seizures, 61% of myoclonic seizures, 64% of generalized tonic-clonic seizures, 69 % of partial seizures, 100 % of tonic seizures, and in 40 % of atonic seizures.

During the study, six children discontinued treatment for severe drowsiness, three children for severe restlessness, and three children for exacerbation of seizures. Mild adverse events were seen in 9 % (N=28) of patients, including drowsiness and somnolence in 10 children, anorexia in eight, fatigue in five, headache in three, and ataxia and diplopia in two children. Side effects of using the drug occurred within the first 2-4 weeks, and such transient complications were resolved with continued use of the drug. No life-threatening side effects were reported.

The frequency distribution of good response to LEV based on some patients' characteristics is shown in Table 1, which indicates that LEV was significantly more effective in partial seizures, idiopathic epilepsies, children with normal developmental status, and normal brain MRI. Also, no statistically significant differences were seen from viewpoints of sex distribution, family history of epilepsy, and mean age of patients in both groups. 

In the responders group, the mean age of onset of seizures was higher than in non-responders. 

## Discussion

This study was designed to evaluate the efficacy, tolerability, and safety of LEV in children with refractory epilepsy in Yazd, Iran. The present research demonstrated that LEV appears to be effective on different types of seizures (generalized, focal, and mixed), which is consistent with the results of preceding studies. ^(^^8^^-^^16^^)^

A meta-analysis study showed that LEV at the dosage of 60 mg/kg/day compared to placebo was a more effective and safer antiepileptic drug in treating generalized or partial intractable epilepsies of children. ^(^^5^^)^

In this research, children aged 1-14 years (mean age: 6.78 ± 2.12 years) were enrolled. However, in studies conducted in Tehran, Iran, children aged 0.6–15 years (median: 5.9 years) ^(^^11^^)^, in France, patients aged 2.1-19 years (mean age: 10.8 years) ^(^^16^^)^, and in Adana, Turkey, children with a mean age 96.00 ± 31.15 months were evaluated ^(^^4^^)^. 

In the present research, the mean dose of LEV for seizure control was 27.9 ± 5.4 mg/kg/day. However, the average control dose was 46.1 mg/kg/day in Japan ^(^^9^^)^, 43.7 ± 20 mg/kg/day in Israel ^(^^12^^)^, and 47 mg/kg/day in Seoul, Korea ^(^^17^^)^.

In this study, at the end of six months of LEV treatment, 20% of epileptic children became seizure-free. Complete seizure control rate was 8.7% in Iran ^(^^11^^)^, 15.6% in Turkey ^(^^4^^)^, 10.3% in China ^(^^8^^)^, 24.6% in Japan ^(^^9^^)^, 44% in Israel ^(^^12^^)^, 23.8% in France, ^(^^16^^)^ and 22% in South Korea ^(^^17^^)^. Also, in this research, a good response (more than 50% reduction in seizures frequency) was seen in 28% of epileptic children. However, 28.3% in Tehran, Iran ^(^^11^^)^, 45.1% in Turkey^ (^^4^^)^, 37%, 24.6%, and 42% in Japanese patients ^(^^6^^,^^9^^, ^^10^^)^, 95% in Israel ^(^^12^^)^, 38.1% in France ^(^^16^^)^, and 48% in South Korea ^(^^17^^) ^achieved more than 50% seizure frequency reduction. 

A possible explanation for these discrepancies is the difference in sample size, age of patients, duration of the study, drug pharmacokinetics, race, and geographic location. 

In the present study, a good response to LEV was more frequent in epileptic children with normal brain MRI. In Japan, 72% (13 of 18 children) of children with a good response (more than 50% reduction in seizures frequency) had normal brain MRI. ^(^^6^^)^

In this research, a good response was seen in 51% of mixed type, 61% of myoclonic seizures, 64% of generalized tonic-clonic seizures, and 69% of partial seizures.

In Turkey, LEV decreased seizure frequency by at least 50% in 58.3% of partial seizures, 32% of generalized seizures, and 17.6% of patients with both partial and generalized seizures.^ (^^4^^)^ In Japan, a 50% reduction in seizures frequency was seen in 42% of localization-related epilepsy and in 35% of generalized epilepsy.^ (^^10^^)^ In France, the efficacy of LEV was similar in partial and generalized epilepsy ^(^^16^^)^. In South Korea, a good response was seen in 52% of partial seizures and in 44% of generalized seizures, and efficacy did not differ significantly among seizure types ^(^^17^^)^.

In this study, during the research period, 12 children (3.8%) discontinued LEV due to severe drowsiness, restlessness, and exacerbation of seizures. However, these adverse events were not serious and life-threatening and were seen in a small number of studied patients. Also, 9% of children had mild and transient side effects, such as somnolence, anorexia, fatigue, headache, ataxia, and diplopia. Frequency of adverse effects was 17% ^(^^16^^)^, 22% ^(^^6^^)^, 30% ^(^^12^^)^, and 37.5% ^(^^18^^)^ in other studies. Dysphoria and mental and behavior disorders occurred in 3% of children in the study by Chen et al.. ^(^^8^^)^. Asthenia, behavioral disorders ^(^^16^^)^, irritability ^(^^5^^,^^10^^)^, and hyperactivity and impulsivity ^(^^10^^)^ were reported in other studies. However, age has been shown to have a significant contribution in experiencing adverse reactions, and the difference in adverse effects observed in these studies can be related to differences in the evaluated age groups by these studies. 

In the present study, the aforementioned adverse effects in the majority of children were transient and mild and did not require the drug discontinuation, and LEV add-on therapy was safe and tolerable in the treatment of refractory epilepsy of children that is consistent with the results of other studies.^ (^^5^^,^^9^^,^^10^^, ^^12^^ , ^^16^^-^^19^^)^

In conclusion, based on the results of this study, LEV was effective in reducing the frequency of seizures in children with refractory epilepsy and can be used as an effective, tolerable, and relatively safe drug in the treatment of generalized, mixed, and partial seizures, as well as various types of epilepsy (idiopathic, cryptogenic, and symptomatic epilepsy). LEV can be suggested as a safe and efficacious modality in the treatment of childhood seizures.

In conclusion, based on the results of this study, levetiracetam was effective in reducing the frequency of seizures in children with refractory epilepsy and can be used as an effective, tolerable and relatively safe drug in the treatment of generalized, mixed and partial seizures, as well as various types of epilepsy (idiopathic, cryptogenic, and symptomatic epilepsy). Levetiracetam can be suggested as a safe and efficacious modality in the treatment of childhood seizures.

**Table.1:** Frequency distribution of good response to levetiracetam based on some patients' characteristics

Response		Good response		P. Value
Yes	No			
Sex	Girl	73 (51%)	69 (49%)	0.8
	Boy	78 (45%)	94 (55%)	
Seizure type	Partial	20 (69%)	9 (31%)	0.03
	Generalized	77(43%)	102 (57%)	
	Partial and generalized	54 (51%)	52 (49%)	
Family history of epilepsy	Yes	91 (48%)	98 (52%)	0.9
	No	60 (48%)	65 (52%)	
Etiologic classification	Symptomatic	48 (35%)	87 (65%)	0.02
	Cryptogenic	58 (51%)	56 (49%)	
	Idiopathic	45 (70%)	20 (30%)	
Developmental status	Delay	112 (46%)	131(54%)	0.02
	Normal	50 (70%)	21(30%)	
MRI findings	Normal	36 (30%)	83 (70%)	0.01
	Abnormal	115(59%)	80(41%)	
Levetiracetam side effects	Yes	13(46%)	15(54%)	0.8
	No	138(48%)	148 (52%)	
Age in year (mean ±SD)		6.26 ± 1.12	5.87 ± 2.54	0.8
Onset age of seizure in year (mean ±SD		2.01± 0.28	0.91± 0.3	0.02

## Autho’s contribution

Dr. Razieh Fallah: Writing the manuscript 

Dr. Alireza Shafiei: Editing the manuscript 

Dr. Fatemah Dehghani Firouzabadi: Gathering the data

Dr. Ali Fathi: Help in writing the manuscript

## Conflicts of interest

The researchers received no financial support from the drug company. The authors declare that there is no conflict of interests.
